# Learning to use working memory: a reinforcement learning gating model of rule acquisition in rats

**DOI:** 10.3389/fncom.2012.00087

**Published:** 2012-10-30

**Authors:** Kevin Lloyd, Nadine Becker, Matthew W. Jones, Rafal Bogacz

**Affiliations:** ^1^Intelligent Systems Laboratory, Department of Computer Science, University of BristolBristol, UK; ^2^School of Physiology and Pharmacology and MRC Centre for Synaptic Plasticity, University of BristolBristol, UK

**Keywords:** working memory, reinforcement learning, gating models

## Abstract

Learning to form appropriate, task-relevant working memory representations is a complex process central to cognition. Gating models frame working memory as a collection of past observations and use reinforcement learning (RL) to solve the problem of when to update these observations. Investigation of how gating models relate to brain and behavior remains, however, at an early stage. The current study sought to explore the ability of simple RL gating models to replicate rule learning behavior in rats. Rats were trained in a maze-based spatial learning task that required animals to make trial-by-trial choices contingent upon their previous experience. Using an abstract version of this task, we tested the ability of two gating algorithms, one based on the Actor-Critic and the other on the State-Action-Reward-State-Action (SARSA) algorithm, to generate behavior consistent with the rats'. Both models produced rule-acquisition behavior consistent with the experimental data, though only the SARSA gating model mirrored faster learning following rule reversal. We also found that both gating models learned multiple strategies in solving the initial task, a property which highlights the multi-agent nature of such models and which is of importance in considering the neural basis of individual differences in behavior.

## 1. Introduction

Working memory involves the short-term maintenance of task-relevant information and is essential in the successful guidance of many behaviors (for review see Baddeley, 2012). However, in facing a new task requiring working memory, it may not initially be clear which information needs to be maintained in memory. Gating architectures (e.g., Braver and Cohen, 1999, 2000; Rougier et al., 2005; O'Reilly and Frank, 2006) model working memory as a collection of past observations and assume that reinforcement learning (RL) shapes useful working memory representations by solving the problem of when to update vs. maintain memory elements. Such models have proved capable of solving challenging memory-based problems such as variants of the *n*-back task (O'Reilly and Frank, 2006) while also displaying learning limitations consistent with working memory limitations found in humans (Todd et al., 2009). Zilli and Hasselmo (2008) recently demonstrated how an RL gating model could perform at above chance level in a range of memory-dependent tasks from the rat experimental literature, including several maze tasks. Investigation into the general properties of such models as well as their ability to speak in detail to real data remains, however, at an early stage. For example, the evidence for one RL algorithm rather than another being implemented in the brain is mixed (Lalonde, 2002; Roesch et al., 2007) and it is likely that different RL methods yield distinct behaviors in gating models. The aim of the current work was to investigate in detail the ability of gating models to match behavioral data by comparing the behavior of two RL gating models with the learning pattern of rats in a rule learning task. We show that both gating models produce behavior consistent with initial rule-acquisition by the animals but differ in their abilities to replicate faster learning following rule reversal. Furthermore, we highlight the ability of both gating models to converge on multiple strategies and relate this property to multi-agent RL (MARL) systems in general.

## 2. Materials and methods

### 2.1. Behavioral task

**Figure 1:**
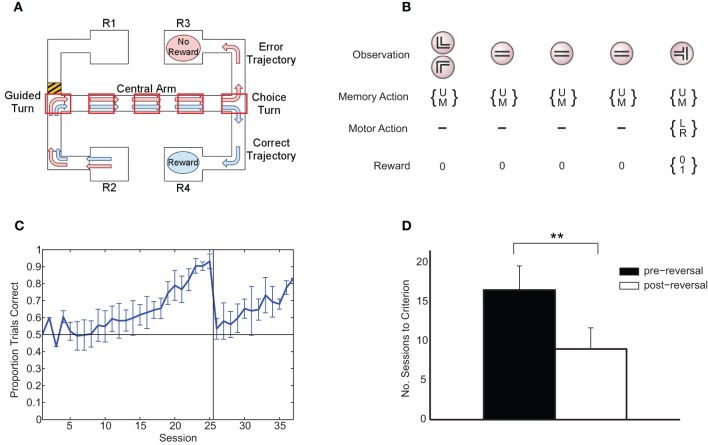
**(A)** Rule-learning maze task. The animal starts at either R1 or R2 and is guided down the central arm at the end of which it must choose to turn left or right. Under the rule depicted here, in order to get a reward, the animal must choose the same direction at the choice turn as at the guided turn (in this case, right). Subsequently (not shown), the animal is guided back to a reward at either R1 or R2 to begin the next trial, with the starting point selected at random from trial to trial. The five boxes superimposed on the central arm of the maze represent an example discretization of the maze in the abstract version of the task presented to the learning algorithm. Modified from Jahans-Price (unpublished). **(B)** Version of task presented to the learning algorithm for the case of *N* = 5 maze points (corresponding to the discretization in panel **A)**. On each trial, an initial left or right guided turn observation is followed by a fixed number of identical “distractor” central-arm observations prior to the final choice-turn observation. The gating architecture chooses to either update (U) or maintain (M) its current memory content on each time step and chooses between left (L) and right (R) turns at the choice turn. Rewards are zero except at the choice turn where reward is either 0 or 1 depending on whether the choice is consistent with the current rule. **(C)** Average fraction of correct choices per session over all animals (±1 standard deviation). On each experimental session, rats ran an average of 36 ± 4 trials, with equal numbers of left and right starts in each session. **(D)** Mean number of sessions to reach 80% performance (+1 standard deviation). ^**^ indicates a significant difference at the 1% level.

We employed a maze task (Jones and Wilson, 2005) in which six (adult, male Long-Evans) rats had to choose between left and right maze arms based on the direction of an initial guided turn (Figure 1A). Animals were initially trained to a criterion level of performance (three sessions of at least 85% of trials correct) under a “match turn” rule (i.e., if initially forced to turn right or started at R2, turn right at the choice point, and vice versa; this is the rule depicted in Figure 1A). The rule was subsequently switched to the corresponding “non-match turn” rule (i.e., if initially forced to turn right or started at R2, turn left at the choice point, and vice versa).

### 2.2. Computational model and architecture

For modeling purposes, we created a highly schematic version of the maze task (Figure 1B). The maze is discretized into a number of distinct locations (boxes, Figure 1A) at which observations from a feature set O = {left turn, right turn, central arm, choice turn} are given depending on current location. The difficulty of the task could be altered by varying the number of intermediate “central arm” points between the guided and the choice turns.

Gating architectures model working memory as a collection of memory elements where past observations may be actively maintained to guide ongoing behaviour. RL is assumed to play a role in shaping useful working memory representations by solving the problem of when to update vs. maintain memory contents. Such models can be viewed as comprising two types of agent: one or more *gating agents*, each of which has a one-to-one correspondence with a memory elements, and a *motor agent*. The gating agents are responsible for choosing to either update or maintain the current content of their respective memory elements, while the motor agent is responsible for overt choices (e.g., direction of motion within a maze). In the current model, we focus on the simplest case where only a single observation is permitted to be stored in memory (i.e., one memory element). The learning problem can be viewed as the simultaneous learning of distinct motor and memory policies (i.e., state-action mappings) by motor and gating agents, respectively (Todd et al., 2009). As such, gating models can be seen as a MARL systems (Busoniu et al., 2008) in which motor and gating agents implicitly attempt to coordinate their behavior so as to maximize reward. These distinct agents share the same state space but map to different actions: motor actions, which guide physical movement in the maze, and memory actions, which act on the memory store. The state space is defined as the product set of possible observations and memory contents S=O×ℳ, where in the current model the set of possible memory contents is defined as the union of a subset of observations and an “empty state”: ℳ = {left turn, right turn, central arm} ∪ {empty}. The “choice turn” observation is not included in ℳ since a trial terminates immediately after action at the choice turn and so the agent never enters a state in which memory contains this observation. The additional “empty” memory state is enforced as the initial memory state at the beginning of each trial.

RL provides a normative framework for addressing the learning problem, but leaves considerable freedom as to which specific methods to employ. In the current work, we compare two different gating algorithms based on two popular RL methods: Actor-Critic (Barto et al., 1983) and SARSA (Rummery and Niranjan, 1994). Briefly [see Sutton and Barto (1998) for details] both of these algorithms attempt to find optimal policies based on estimating values (expected returns) using temporal difference (TD) methods. SARSA estimates the values of state-action pairs, while the Actor-Critic independently learns and represents both the values of states (the “critic”) and a separate policy structure summarizing action preferences in each state (the “actor”). Since both algorithms have the same overall form, we describe a single iteration of each algorithm in parallel while highlighting the differences. The general steps of both gating algorithms are summarized in Table 1.

**Table 1:** **General steps of the gating algorithms (see text for details)**.

1	Choose motor action *a* and gating action *g* for current state *s* according to softmax over relevant action values in *Q*^*M*^ and *Q*^*G*^ (Equations 1, 2)
2	Observe reward *r* and next state *s*′
3	Compute TD errors (Equations 3–5)
4	Update specific eligibility traces associated with current state *s* and actions *a*, *g* (Equations 6–8)
5	Update all state/state-action values (Equations 9–13)
6	Update all eligibility traces (Equations 14–16)
7	Repeat steps 1–6 until termination

On each time step, a motor action *a* ∈ {go straight, turn left, turn} is chosen by the motor agent on the basis of state-action values $Q^{M}(s,\tilde{a}),$, where $\tilde{a}$ ranges over all possible motor actions and *s* denotes the current state. Similarly, a gating action *g* ∈ {update, maintain} is chosen on each time step by the gating agent on the basis of state-action values $Q^{G}(s,\tilde{g})$. Action selection in both cases is according to the softmax selection rule so that the probabilities of selecting particular actions *a* and *g* in current state *s* are given by

(1) $$P(s,a)=\frac{\exp\{Q^{M}(s,a)/T\}}{\sum_{\tilde{a}}\exp\{Q^{M}(s,\tilde{a})/T\}}$$

(2) $$P(s,g)=\frac{\exp\{Q^{G}(s,g)/T\}}{\sum_{\tilde{g}}\exp\{Q^{G}(s,\tilde{g})/T\}}$$

where the exploration or “temperature” parameter *T* controls the degree of stochasticity in selection such that lowering the *T* value leads increasingly to deterministic choice of actions with higher action values whereas increasing *T* leads increasingly to indifference between actions. Note that for the motor agent, the set of possible motor actions $A_{\text{motor}}$ ⊂ {go straight, turn left, turn right} was specified to reflect the movements of the rats through the maze. Prior to reaching the choice turn, the only available motor action is to “go straight” along the central arm (indicated by a dash in Figure 1B) and so the motor agent simply chooses between turning left or right at the choice turn.

Having performed motor and gating actions, a reward *r* ∈ {0,1} and next state *s*′ are observed. Rewards for all time steps were zero except for correct choices made at the choice turn. As already mentioned, both Actor-Critic and SARSA gating algorithms are based on estimating state and/or state-action values via TDs. However, the TD errors computed in each case differ. For the Actor-Critic version, there is a single TD error δ which is computed on the basis of successive state values:

(3) $$\delta \leftarrow r+\gamma V(s^{\prime })-V(s),$$

where γ is the discount rate which is always set to 1 due to the episodic nature of the task. By contrast, the SARSA gating algorithm computes two TD errors, δ_*M*_ and δ_*G*_, based on the discrepancy between successive state-action values for motor and gating agents, respectively:

(4) $$\delta_{M}\leftarrow r+\gamma Q^{M}(s^{\prime },a^{\prime })-Q^{M}(s,a)$$

(5) $$\delta_{G}\leftarrow r+\gamma Q^{G}(s^{\prime },g^{\prime })-Q^{G}(s,g).$$

Note that computing the TD errors for the SARSA gating algorithm in Equations (4) and (5) requires that the next actions *a*′ and *g*′ have already been chosen [again, via Equations (1) and (2)].

As in previous work (Zilli and Hasselmo, 2008; Todd et al., 2009), we make use of *eligibility traces* (Sutton, 1988) which have been found to be especially useful in partially-observable environments. Eligibility traces can be interpreted as memories or “tags” for the occurrence of states or state-action pairs which allow the values of past states/state-actions to be affected by the current TD error. The amount that the current TD error affects the value of a previous state/state-action depends on the current strength of that state/state-action's trace. How the strength of a trace decays over time is controlled by a decay parameter 0 ≤ λ ≤ 1, where λ = 0 yields decay to zero after a single time step and λ = 1 leads to no decay. In the current model, all traces are initialized to zero at the start of each trial. Following calculation of the TD error, the various eligibility traces associated with the previous state *s* and actions *a*, *g* are updated to record their recent occurrence. For both Actor-Critic and SARSA, this involves setting

(6) $$e^{M}(s,a)\leftarrow 1$$

(7) $$e^{G}(s,g)\leftarrow 1,$$

and for the Actor-Critic, the additional update

(8) $$e^{V}(s)\leftarrow 1.$$

The updating of traces to 1 in this manner corresponds to what is called a “replacing trace” (Sutton and Barto, 1998). Other types of eligibility trace can be used [e.g., an “accumulating trace,” see Sutton and Barto (1998)], but our experimentations with different types of trace suggest that results in the current task are not strongly affected by choice of trace.

All states and/or state-action pairs are now updated in accordance with the TD errors and eligibility trace values. Note that due to the different TD errors computed in Equations (3–5), updates differ for SARSA and Actor-Critic. For SARSA, the updates are

(9) $$\forall s,a:Q^{M}(s,a)\leftarrow Q^{M}(s,a)+\alpha \delta_{M}e^{M}$$

(10) $$\forall s,g:Q^{G}(s,g)\leftarrow Q^{G}(s,g)+\alpha \delta_{G}e^{G},$$

where α is the learning rate, and the agent-specific TD errors δ_*M*_ and δ_*G*_ are used to update motor and gating state-action values, respectively. By contrast, the updates for the Actor-Critic all involve the single TD error δ:

(11) $$\forall s,a:Q^{M}(s,a)\leftarrow Q^{M}(s,a)+\alpha \delta e^{M}$$

(12) $$\forall s,g:Q^{G}(s,g)\leftarrow Q^{G}(s,g)+\alpha \delta e^{G},$$

with the additional state value update

(13) $$\forall s:V(s)\leftarrow V(s)+\alpha \delta e^{V}.$$

The final step of the iteration is to update the eligibility traces of all states and/or state-action pairs by multiplying with the eligibility trace decay λ. For both SARSA and Actor-Critic, one updates

(14) $$\forall s,a:e^{M}(s,a)\leftarrow \gamma \lambda e^{M}(s,a)$$

(15) $$\forall s,g:e^{G}(s,g)\leftarrow \gamma \lambda e^{G}(s,g),$$

with the additional update for the Actor-Critic

(16) $$\forall s:e^{V}(s)\leftarrow \gamma \lambda e^{V}(s).$$

### 2.3. Model fitting

Model parameters were fit to the pre-reversal rat data only. The distribution over the number of sessions to criterion performance (at least 85% of trials correct for three sessions) for the rats was taken as the target distribution and assumed to be Gaussian (no. sessions: 17, 19, 20, 21, 22, 25; $\hat{\mu }$ = 20.7, ${\hat{\sigma }}^{2}$ = 7.5). The model has four free parameters: number of maze points *n*, learning rate α, exploration rate *T*, and the eligibility trace decay λ. For a particular setting of the parameters, a distribution over the number of sessions to criterion performance was obtained from the sample mean and variance of 10,000 simulation runs and the Kullback–Leibler (KL) divergence between the target distribution and simulation distribution measured. Best-fitting model parameters for a given number of maze points *n* was obtained by minimizing the KL divergence using the Nelder–Mead method (Nelder and Mead, 1965) [for more precise details, see Bogacz and Cohen (2004)]. The optimization procedure was carried out several times with different initial parameter values for a given *n* to avoid problems with local minima. For different values of *n*, the parameters associated with the lowest KL divergence were recorded.

## 3. Results

### 3.1. Behavioral data

Six adult rats were trained in a rule-learning maze task (Jones and Wilson, 2005) running an average of 36 (±4) trials per session in which they had to choose between left and right maze arms based on the direction of an initial guided turn (Figure 1A). The average learning curve consists of an initial “pre-reversal” curve (sessions 1–25), showing learning under the match-to-turn rule, and a “post-reversal” curve (sessions 26–37), reflecting learning under the non-match-to-turn rule (Figure 1C). Each rat took at least 17 sessions (≈612 trials) to reach criterion performance on the initial match rule. All rats learned more quickly in the post-reversal phase, taking fewer sessions to reach the same level of performance [*t*_(5)_ = 8.5, *p* < 0.01; Figure 1D].

### 3.2. Rule acquisition is reproduced in gating models

**Figure 2:**
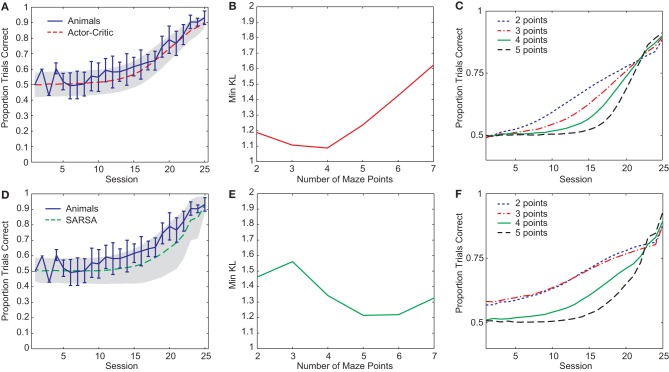
**(A)** Best-fit average learning curve (10,000 simulation runs) during the pre-reversal phase for Actor-Critic gating algorithm (*N* = 4, α = 0.14, *T* = 1.38, and λ = 0.93) compared with average animal learning curve (±1 standard deviation). Shading indicates ±1 standard deviation for simulations. **(B)** Minimum KL divergences for Actor-Critic for different values of *n* (10 runs of optimization algorithm for each *n*). **(C)** Average learning curves (10,000 simulation runs) for Actor-Critic corresponding to best-fit parameters found for different values of *n*. **(D)** Best-fit average learning curve (10,000 simulation runs) during the pre-reversal phase for SARSA gating algorithm (*N* = 5, α = 0.31, *T* = 0.13, and λ = 0.03). **(E)** Minimum KL divergences for SARSA for different values of *n* (10 runs of optimization algorithm for each *n*). **(F)** Average learning curves (10,000 simulation runs) for SARSA corresponding to best-fit parameters found for different values of *n*.

Two RL gating algorithms, one based on Actor-Critic methods (Barto et al., 1983) and the other on SARSA (Rummery and Niranjan, 1994), were given an abstract version of the rule-learning maze task (Figure 1B) and parameters fit to a subset of the rat behavioral data (see “Materials and Methods”). Both gating models yielded reasonable fits to the pre-reversal data (Figures 2A,D) though the Actor-Critic version showed learning more closely resembling that of the rats in terms of average trend and level of variability. In both cases, quality of fit showed a dependence on the number of central arm (“distractor”) observations *n* between guided and choice turns (Figures 2B,E). This dependence on *n* is due to different possible rates of performance improvement for different values of *n* such that small values lead to improvements which are too fast while large values lead to improvements that are too slow compared to the rats' learning (Figures 2C,F).

### 3.3. Gating models generate different strategies

**Figure 3:**
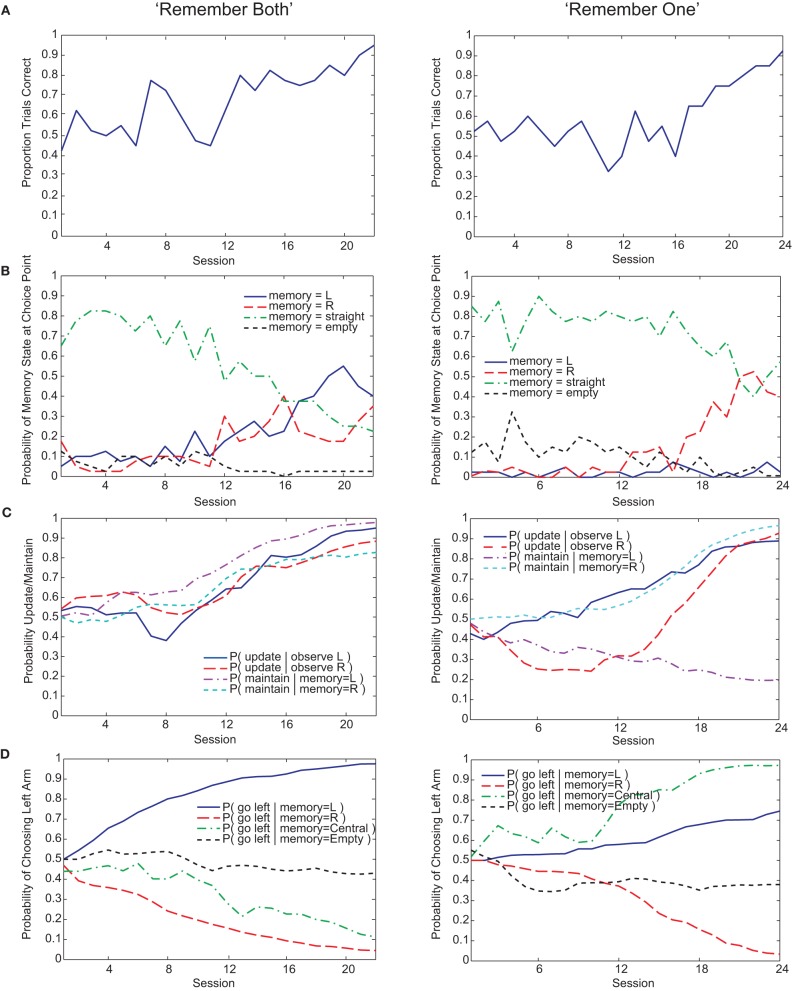
**Examples of “remember both” (left) and “remember one” (right) strategies learned by the Actor-Critic gating model. (A)** Fraction of trials correct per session (40 trials per session). **(B)** Probability over sessions of the different possible memory contents at the choice point. Note that since, on average, 50% of trials begin with “left turn” observations and the other 50% with “right turn” observations, the maximum proportion of a session's trials that either of these observations can be in memory at the choice point is roughly 50%. The qualification “roughly” is due to our selecting of initial observations at random rather than strictly enforcing a 50–50 within a session, in contrast to the experimental procedure for the animals. **(C)** Probability over sessions of updating or maintaining memory conditional on observing the initial guided turn or having this feature already in memory. Probabilities are derived directly from the action values at the end of each session. **(D)** Probability over sessions of choosing to turn left at the choice point as a function of the different possible memory contents. Again, probabilities are derived directly from the action values at the end of each session.

**Figure 4:**
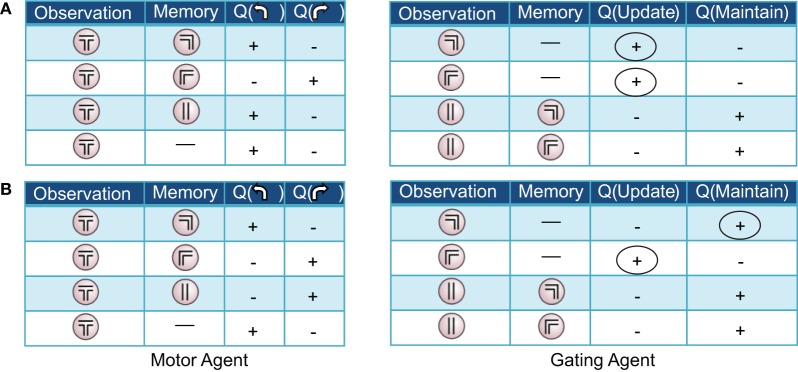
**Two examples of (partial) action-value tables resulting from the pre-reversal phase of learning in the Actor-Critic gating algorithm with best-fit parameters.** For clarity, only the signs of the learned values, positive or negative, are displayed. At the beginning of each trial, the memory is empty (denoted by a dash). **(A)** “Remember both” strategy. The gating agent (right) favors loading/updating the initial observation of right or left guided turn as indicated by positive values for this action (first two rows, circled). During the central arm section of the maze, maintaining the initial observed turn in memory is favored (last two rows). **(B)** “Remember one” strategy. The gating agent (right) favors loading/updating only one of the initial observations (in this case, guided right turn; circled), remaining empty in the other case. In both examples, the motor agent (left tables) tends to choose (correctly) to turn left or right when the left and right guided turn observations are in memory, respectively.

**Figure 5:**
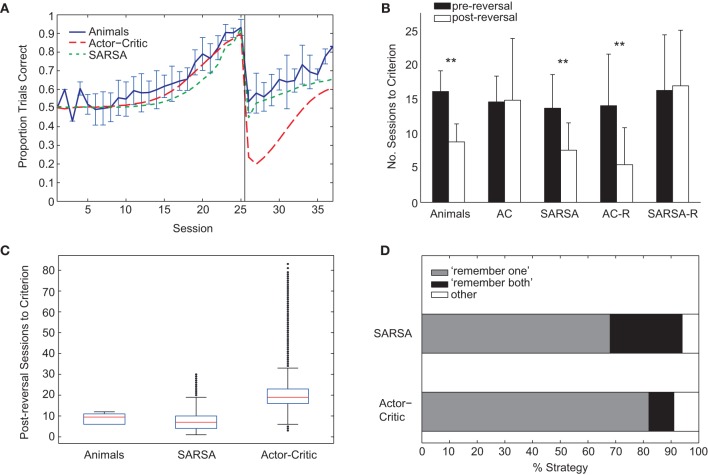
**(A)** Comparison of average learning curves for both pre- and post-reversal phases: animals (blue), Actor-Critic (red, long dash), and SARSA (green, short dash). Simulated learning curves are obtained by averaging over 10,000 runs with parameters fitted only on the pre-reversal data. **(B)** Mean number of sessions to reach criterion performance (80% of trials correct in a session) pre-reversal (black) and post-reversal (white). Error bars indicate one standard deviation. “AC” (Actor-Critic). ^**^ indicates a significant difference in the number of pre- and post-reversal sessions at the 1% level. **(C)** Box plots showing distributions of number of sessions required to reach post-reversal criterion. For each box, the central red mark indicates the median, and the lower and upper edges indicate the lower and upper quartiles (i.e., 25th and 75th percentiles), respectively. Whiskers extend to the lowest and highest data points that fall within 1.5 IQR of the lower and upper quartiles, respectively. Outliers marked as black dots. **(D)** Percentage of strategy types learned by the Actor-Critic and SARSA gating models. Each algorithm was run 100 times, and classification of strategy type was on the basis of the action values on reaching criterion for the pre-reversal phase.

Both Actor-Critic and SARSA gating algorithms found multiple solutions to the pre-reversal task despite their parameters being fixed to best-fit values (Figure 5C). In what we call a “remember both” strategy (Figure 3, left), the algorithm learns to update and maintain memory with the initial guided turn observation, whether left or right. This is reflected in the memory content at the choice point gradually changing from being mostly the “central arm” observation to being almost entirely the initial “left turn” or “right turn” observations by the end of acquisition (Figure 3B, left). It is also reflected by the increasing probability over sessions of loading the initial observation into memory, *P*(update|·), and the probability of maintaining that initial observation if loaded, *P*(maintain|·) (Figure 3C, left). As expected, the motor agent learns to choose the left arm when the initial “left turn” observation is present in memory, and the right arm when the initial “right turn” observation is present in memory (Figure 3D, left). The “remember both” strategy is also reflected in the pattern of learned action values (Figure 4A).

By contrast, a “remember one” strategy only remembers the initial observation for either a left or right guided turn (Figure 3, right, and Figure 4B). Thus, the probability of memory containing the initial turn will only increase over time for one of the initial turn directions, for example a “right turn” (Figure 3B, right). In the specific example shown, while the probability of loading an initial “left turn” observation increases, the probability of maintaining such an observation in memory actually decreases (Figure 3C, right). This strategy is viable because having the “central arm” observation in memory at the choice point is strongly indicative of having initially observed a left guided turn, and the motor agent learns to respond accordingly (Figure 3D, right).

For both SARSA and Actor-Critic gating algorithms, the “remember one” strategy was more common, occurring on approximately 70% and 80% of simulation runs, respectively (Figure 5D). The relative frequencies of different kinds of strategy were found by running fitted models 100 times and classifying the resultant strategy as either “remember both” or “remember one” on the basis of the proportion of final-session trials in which the direction of the initial guided turn was in memory at the choice point. In particular, a threshold was used: if the proportion of left guided trials with “left turn” in memory at the choice point was above two-thirds, this counted as a “remember left” strategy, and vice versa; if proportions for both “left turn” and “right turn” were above threshold, this was counted as “remember both”; finally, if the threshold was not reached for either “left turn” or “right turn” trials, the classification was “other.”

### 3.4. Actor-critic and sarsa differ in modeling transfer of learning

Unlike the rats, the Actor-Critic gating architecture did not show positive transfer of learning with performance dropping well below chance following reversal (Figure 5A) and the mean number of sessions required to reach criterion performance being approximately equal for pre- and post-reversal learning phases (Figure 5B, “AC”). In contrast, the performance of the SARSA gating algorithm fell to chance level following rule reversal and learning proceeded faster for the reversed rule (Figures 5A,B) in accordance with the experimental data, though the comparatively shallow slope of the SARSA average learning curve over the post-reversal period reflects a difference in individual learning curve variability compared to the rats. In particular, a substantial number of SARSA simulation runs take longer than 12 sessions to reach the post-reversal criterion, the greatest number of sessions required by the rats (Figure 5C).

**Figure 6:**
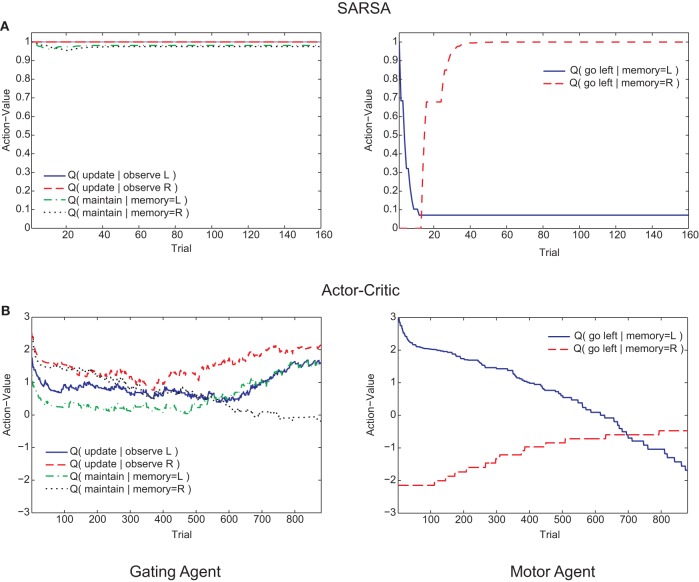
**Examples of trial-by-trial changes in action values of the memory agent (left graphs) and motor agent (right graphs) during the post-reversal phase. (A)** SARSA gating algorithm. **(B)** Actor-Critic gating algorithm.

Examining the course of learning during the post-reversal phase clarifies the distinct behaviors of the two algorithms. With SARSA, the memory policy learned during the pre-reversal phase is maintained as the motor policy rapidly adapts (Figure 6A). By contrast, with the Actor-Critic, the motor policy adapts on a much slower time scale and the action values for the gating agent are destabilized (Figure 6B). It can be seen that the action values of the Actor-Critic gating algorithm, unlike those for SARSA, are not bounded between 0 and 1 (c.f. Bogacz and Larsen, 2011). Thus, the amount by which the motor action values need to adapt in order to produce a reversal is greater than if they were bounded to the 0–1 interval. This fact is related to the absence of positive transfer, confirmed by modifying the Actor-Critic so that action values are bounded between 0 and 1. In this restricted version, positive transfer is also observed (Figure 5B, “AC-R”).

## 4. Discussion

The current study explored the ability of simple RL gating models, one based on the Actor-Critic and the other on SARSA, to replicate rule learning behavior in rats. Rats were trained in a maze-based spatial learning task that required animals to make trial-by-trial choices based on previous experience. Parameter-fitting of Actor-Critic and SARSA gating algorithms to a subset of the behavioral data produced rule-acquisition behavior consistent with the experimental data for both algorithms. Surprisingly, the SARSA gating model mirrored the faster learning of the rats following rule reversal, an effect also generated by a modified version of the Actor-Critic. Furthermore, both gating models learned multiple strategies in solving the initial task, a property which highlights the multi-agent nature of such models. In the remainder, we discuss the implications of each of these findings in more detail.

### 4.1. Task difficulty and the rate of learning

During the pre-reversal phase, animals took over 600 trials to reach criterion performance. This apparent difficulty in learning the task was consistent with the behavior of RL gating models faced with the problem of simultaneously learning valid motor and gating policies. Reproducing the rats' pre-reversal behavior depended on varying the number of “distractor” observations *n* between the initial guided turn observation and the choice turn in the model task. *n* affects the probability of maintaining the initial observation in memory: as *n* increases, this probability decreases geometrically and it becomes less likely for the initial observation to be in memory, and therefore increasingly difficult to learn a valid policy. Increasing the number of memory elements beyond some small number does not necessarily overcome this difficulty due to the size of the state space scaling exponentially with the number of such elements, making value-learning increasingly difficult (Todd et al., 2009). Although it is not possible to categorically equate the parameter *n* to task or psychological variables, the most natural interpretation of *n* is as an effective baseline forgetting rate which may be reflected in limitations of the rats' working memory for at least some types of information (Baird et al., 2004; Futter and Aggleton, 2006). “Baseline” denotes that the probability of replacing an item in working memory is determined not only by the number of memory actions required in a trial but also by the current action values which change over time. *n* may also be interpretable in relation to the difficulty of inferring which features of the task are relevant to predicting reward (Restle, 1957). However, other factors are likely to have contributed to the difficulty of the task for the animals which we have not explicitly modeled here. For example, rats have a well known natural propensity to spontaneously alternate their choices (Lalonde, 2002) which would clearly interfere with learning the current task.

### 4.2. Transfer of learning

Positive transfer of learning was illustrated by the animals taking fewer trials to reach criterion performance when the original rule was reversed. Whilst the Actor-Critic gating algorithm failed to show positive transfer, the success of the SARSA gating algorithm (and a modified Actor-Critic) in replicating this effect appeared to rely on the stability of the memory policy. This memory policy stability contrasts with the rapid adaptation of the motor policy at the choice turn, an effect ultimately explained by differences in the time scales of learning in relation to the structure of the maze task. The motor actions immediately precede the binary-valued reward of each trial, whereas most of the memory actions take place earlier in the trial (memory actions are also taken at the choice point but are unrelated to reward). This means that the motor agent eligibility traces are generally larger at this point during the task (unless there is no eligibility trace decay, i.e., λ = 1; for the SARSA gating algorithm, the best-fit trace decay was λ = 0.03, a very rapid decay). When the experimental rule is reversed, the TD errors arising at the end of the trial therefore drive greatest changes in the motor agent action values. If the motor policy adjusts sufficiently quickly, the memory policy will be minimally disrupted. Positive transfer therefore arises due to the different timescales of learning of the motor and gating agents. Support for this explanation was obtained by fitting the SARSA gating algorithm to the pre-reversal data while restricting the eligibility trace decay to be λ = 1 (i.e., no eligibility trace decay within a trial). In this case, no positive transfer was obtained (Figure 5B, “SARSA-R”). It should be highlighted that these different timescales arise not because of differences in parameterization of the motor and gating agents (the learning rate parameter was the same for both), but as an “emergent effect” of the workings of the algorithm and structure of the task.

We should not expect such emergent effects to be useful in general. The basic gating architecture shares with other simple model-free RL algorithms the serious limitation of being unable to store multiple policies/rules since only one value function is learned over time. This means that the same set of values are updated continuously as tasks change, leading to maladaptive forgetting in environments where it would clearly be advantageous to recall previously-learned task knowledge when the same or similar tasks arise. For example, in serial reversal learning, while animals show increasingly rapid switching of behavior when faced with multiple reversals of reinforcement contingencies [up to perfect switching between rules following a single trial, e.g., Dufort et al. (1954)], such behavior cannot be produced by the models considered here. Recently, Dayan (2007, 2008) has proposed a uniform gating architecture which is able to instantiate different rules depending on an associative rule-retrieval and rule-matching process. This more flexible system raises issues of how to match rules, when to form new rules, how to transfer knowledge between rules, and other issues beyond the scope of the current discussion, but Dayan's proposals clearly provide a promising framework for future work.

### 4.3. Mixed strategies: individual biases and multi-agent learning

Both Actor-Critic and SARSA gating algorithms learned different viable memory strategies for learning to behave consistently with the original task rule, with a “remember one” gating strategy being more commonly learned in both cases (Figure 5C). The prevalence of this gating strategy as a learning outcome can partly be explained by there being two viable “remember one” strategies and only one possible “remember both” strategy that can solve the task. It is well known that rats can make use of different information, such as allocentric vs. egocentric (Restle, 1957) and retrospective vs. prospective (e.g., Ferbinteanu and Shapiro, 2003), in solving maze tasks, which we did not control for in our experiments. However, the models' learning of multiple strategies suggests a further possible source of variability, namely differences in how such information is used to guide action. This possibility is relevant to studies of neuronal mechanisms underlying learning in such tasks. More generally, the learning behavior of the algorithms highlights the nature of MARL algorithms (Busoniu et al., 2008). We presented the gating algorithms as comprising multiple RL agents, implicitly attempting to coordinate their actions so as to maximize reward. This multi-agent perspective immediately brings into consideration key issues in the MARL field such as the stability of agents' learning dynamics and the adaptation of each agent's behavior to the changing behavior of other agents. From this perspective, the existence of multiple behavioral equilibria in a multi-agent system, exemplified by the learning of different possible strategies in the present case, is not unexpected. However, these issues have not been sufficiently considered in relation to the gating framework.

## 5. Concluding remarks

The development of gating models able to accurately recapitulate learning behaviors is an important prerequisite to using the gating framework to provide insight into the neural structures and mechanisms that support cognitive processing. The present work takes steps in this direction by testing the ability of such models to speak to behavioral data in a detailed manner. In doing so, we highlighted non-trivial properties of gating models such as their convergence to different solutions and differences of behavior resulting from alternative choices of learning algorithm. With regard to the latter, our results suggest the use of SARSA or modified Actor-Critic in reproducing faster learning following rule reversal in simple gating models. More generally, our results suggest that choice of RL algorithm is an important consideration in the use of gating models, making the question of which algorithms are biologically instantiated all the more pressing. The approach is therefore likely to prove particularly important when applied to tasks readily combined with the monitoring of neural network activity, including maze-based tasks in rodents of the sort considered here.

Turning to questions of biological implementation, most models of working memory assume implementation by ensembles of neurons able to stably maintain a pattern of activity over time (e.g., Wang, 1999). In their model of working memory based on the prefrontal cortex and basal ganglia, O'Reilly and Frank (2006) propose a neural implementation of an Actor-Critic algorithm in which patterns are maintained in the prefrontal cortex under the control of the basal ganglia, where the latter (along with the midbrain and amygdala) learns both which prefrontal representations are relevant (critic) and a gating policy controlling working memory updating (actor). Relating to the present study, neurons in the hippocampus and medial prefrontal cortex of rats trained in the current task tend to fire at higher rates in the central arm depending on the direction of the initial guided turn (Jones and Wilson, 2005). However, these neurons did not fire uniformly throughout the central arm but rather showed preference for certain central arm locations. Relatedly, Harvey et al. (2012) found that distinct sequences of posterior parietal cortex neurons were triggered depending on behavioral choice when mice were presented with a similar memory-based task in a virtual environment. Such findings suggest that working memory in maze tasks may be encoded in sequence-based circuit dynamics rather than long duration stable states. Extending the current model to explicitly encode spatial position will therefore be crucial to relating model behavior to neuroscientific findings.

### Conflict of interest statement

The authors declare that the research was conducted in the absence of any commercial or financial relationships that could be construed as a potential conflict of interest.
